# Characterization of Occupational Exposure To Fungal Burden in Portuguese Bakeries

**DOI:** 10.3390/microorganisms7080234

**Published:** 2019-08-02

**Authors:** Carla Viegas, Tiago Faria, Liliana Aranha Caetano, Elisabete Carolino, Anita Quintal-Gomes, Magdalena Twarużek, Robert Kosicki, Susana Viegas

**Affiliations:** 1 Instituto Politécnico de Lisboa, H&TRC―Health & Technology Research Center, ESTeSL―Escola Superior de Tecnologia da Saúde, 1990-096 Lisboa, Portugal; 2 Centro de Investigação em Saúde Pública, Escola Nacional de Saúde Pública, Universidade NOVA de Lisboa, 1600-560 Lisboa, Portugal; 3 Centro de Ciências e Tecnologias Nucleares, Instituto Superior Técnico, Universidade de Lisboa, E.N. 10 ao km 139,7, 2695-066 Bobadela LRS, Portugal; 4 Research Institute for Medicines (iMed.ULisboa), Faculty of Pharmacy, University of Lisbon, 1649-003 Lisbon, Portugal; 5 Faculty of Medicine, Institute of Molecular Medicine, University of Lisbon, 1649-028 Lisbon, Portugal; 6 Faculty of Natural Sciences, Department of Physiology and Toxicology, Institute of Experimental BiologyKazimierz Wielki University, Chodkiewicza 30, 85–064 Bydgoszcz, Poland

**Keywords:** bakeries, fungal contamination, mycotoxins contamination, occupational exposure, multi-approach analyses

## Abstract

Several studies reported adverse respiratory health effects in workers exposed to ambient contaminants in bakeries. The aim of this study was to examine worker exposure to fungi and mycotoxins in Portuguese bakeries in order to develop new policies in occupational health. Environmental samples such as air, surfaces, settled dust and electrostatic dust collector (EDC) were collected in 13 bakeries for fungal and mycotoxins assessment. Air samples obtained by impaction were performed applying malt extract agar (MEA) supplemented with chloramphenicol (0.05%) and dichloran glycerol (DG18) agar-based media. Air samples collected through impinger method were determined as well for fungal detection by molecular tools of *Aspergillus* sections and mycotoxins. The highest median value for fungal load was 1053 CFU·m^−3^ and 65.3% (32 out of 49) of the sampling sites displayed higher fungal load than limits imposed by the World Health Organization. *Aspergillus* genera was found in air, surface swabs and EDC. Molecular tools were effective in measuring *Aspergillus* section *Fumigati* in 22.4% on air, 27.8% on surface swabs and in 7.4% in EDC and *Aspergillus* section *Versicolores* in one air sample. All settled dust samples showed contamination with six to eight mycotoxins in each sample. The mycotoxins detected were deoxynivalenol-3-glucoside, deoxynivalenol, zearalenone, 15-acetyldeoxynivalenol, monoacetoxyscirpenol, diacetoxyscirpenol, fumonisin B1, fumonisin B2, griseofulvin, HT2, ochratoxin A, ochratoxin B and mycophenolic acid. Industrial hygienists and exposure assessors should rely on different sampling methods (active and passive) and different assays (culture based and molecular methods) to obtain an accurate risk characterization regarding fungal burden (fungi and mycotoxins). Additionally, the awareness for the raw material as a potential mycotoxins indoor contamination source is important.

## 1. Introduction

Baking is a major industrial activity in Portugal and directly relates to the fact that Portuguese bread is a well-known product that is appreciated both nationally and internationally [1]. However, without precise numbers, this implies a considerable work force involving many workers in this specific occupational environment in Portugal.

In this setting ‘baker’s asthma’ is one of the most common work-related respiratory diseases. Wheat sensitization prevalence rates of up to 30% have been reported for bakers [2]. The environment of bakeries is very complex, and has several potential sensitizers among cereal flours such as wheat, rye or barley. However, there are reports of baker’s asthma caused by fungi, yeast, eggs, sesame seeds, nuts, and insects as well. The occurrence of sensitization to these allergens is less well known than those cases caused by cereal flours or enzymes, but they should be kept in mind in the clinical setting if no sensitization to common bakery allergens is found [3].

Several occupational exposure limits to flour dust were proposed as follows: (1) threshold limit value of 0.5 mg/m^3^ for flour dust in flour mills [4] by the American Conference of Governmental Industrial Hygienists (ACGIH); (2) 10 mg/m^3^ total dust and 5 mg/m^3^ for respirable dust in certain Canadian provinces [5]; (3) 4 and 3 mg/m^3^ for flour dust in Germany and Denmark, respectively; (4) 5 mg/m^3^ for organic dust in Finland, Iceland, and Norway [6]; and (5) 8 h TWA maximum exposure limit of 10 mg/m^3^ for flour dust, with a 15 min exposure limit of 30 mg/m^3^ by the health and safety commission of the United Kingdom [7]. Thus, differences exist in the exposure limits even within the same continent. 

Considering the exposure limits, it is possible to recognize that the quantitative characterization of flour dust and allergens is based upon air or settled dust sampling [8]. However, flour is a complex organic dust encompassing one or a mixture of different cereal grains including wheat, rye, millet, barley, oats or corn cereal that have been processed or grounded by milling [9]. In addition, flour may contain several contaminants, such as fungi and mycotoxins [5,8], being the raw materials (such as different cereals and flour) entering the facilities the principal contamination sources for this occupational environment―as is the case in other settings where fungal burden was assessed [10]. 

Centered on the toxigenicity of several mycotoxins, regulatory levels have been established to food commodities by many national governments and adopted for use in national and international food trade. Internationally, Codex, the European Union (EU), and other regional organizations issued a number of decrees recommending maximal levels of mycotoxins in foods and feeds, which were used as guidelines for controlling contamination by mycotoxins for consumer safety [11]. In France, measurements in different food commodities—such as cereals, vegetables, and spices—showed no contamination above regulatory limits. However, workers’ exposure via inhalation was high for all settings as the air measurements revealed significant levels of mycotoxins bound to dust particles [12]. Previously, Viegas et al. [13,14,15,16] demonstrated that occupational exposure to mycotoxins occurs essentially by inhalation. Recently, Viegas et al. [17] demonstrated that inhalation of mycotoxins on the workplace significantly adds to the exposure resulting from ingestion of mycotoxin-contaminated food.

The highest levels of exposure to organic dust occur in two different phases (mixing and baking) in both small and large bakeries and upon receiving and opening of flour containers in larger bakeries [18]. Several studies reported adverse respiratory health effects in workers exposed both in small and large-scale industries [8,19,20]. Indeed, adverse respiratory system symptoms and diseases were found to be induced by occupational dust being influenced by the type of dust, dose, duration of exposure and genetic factors. 

Different sampling methods should be applied to ensure a more detailed occupational exposure assessment to fungal burden as each method has unique advantages and disadvantages. Thus, a multi-approach in the sampling methods will enrich data findings, therefore enabling a more accurate risk characterization [10,21,22]. 

Until now, data covering a wider spectrum of the fungal burden (fungi characterization and mycotoxins presence), obtained by a multi-approach on sampling methods and assays in bakeries have not been reported, and this omission has barred the implementation of suitable preventive measures. This is of particular importance as “baker’s asthma” can be caused by fungi as well.

The aim of this study was to assess workers exposure to fungi and mycotoxins in Portuguese bakeries in order to develop new policies in occupational health.

## 2. Materials and Methods

### 2.1. Bakeries Characteristics and Samples collection 

The study was conducted between November 2016 and June 2017 in 13 Portuguese bakeries located in the Lisbon district. The bakery samples were obtained through random selection of the bakeries covered in this study, which belong to two different companies that accepted to participate. Eight bakeries belonged to a large company producing bread for sale in their own stores as well as for different school canteens and vending machines. The other five bakeries were integrated into supermarket facilities and belonged to a supermarket representative of a large group of 292 units widespread in Portugal with similar features concerning the bakery section (Table 1).

Most of the units were divided into three different areas which consisted of the sampling sites considered for this study: production—where kneading machines and ovens were located and where dough shaping was performed; raw material warehouse—where workers need to go several times to collect raw materials for dough preparation; store—where the final product is sold (bread or pastry). These areas correspond with the workplaces where the workers spend more time as well. In one of the bakeries without store a distinct area was assessed: expedition—where distribution of final product for other units occurs. Further, one bakery was only devoted to pastry. It is important to mention that normally workers involved in bread production spend most of their time in the production area and, occasionally—depending upon production rate—when they need to produce new dough, they go to the raw material warehouse where they only spend a few minutes. The production workers normally do not spend time in the store as this workplace has dedicated workers that remain in the store.

The sampling sites and collection periods (depending upon the equipment and assay applied) for each bakery were determined based upon the significant amount of time spent by workers in those places during their occupational activity and the tasks developed in each workplace. In these workstations, environmental samples including air, surfaces, settled dust and electrostatic dust collector (EDC) were obtained for the assessment of fungal burden (fungi and mycotoxins) and sampling performed during a normal working day (Table 1). 

### 2.2. Sampling Approaches Characterization

**Figure 1:**
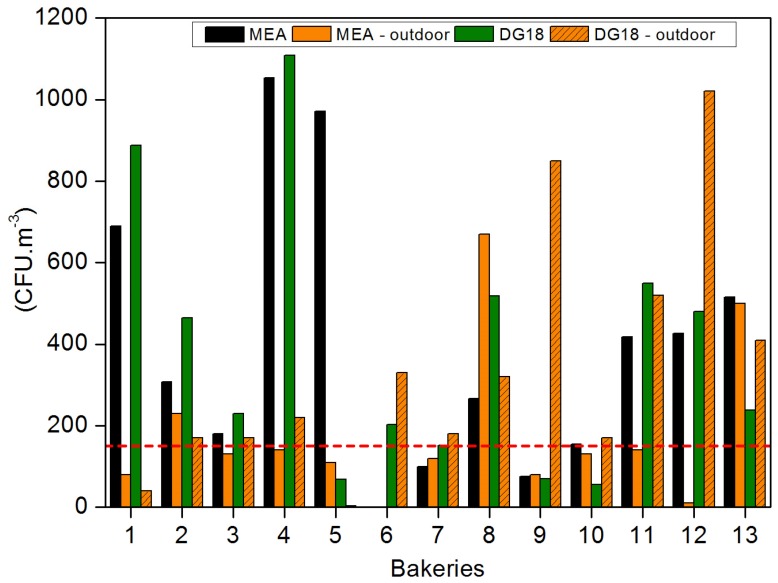
Air fungal load distribution in the 13 assessed bakeries. The dashed line represents the reference limits suggested by the World Health Organization (WHO).

Air samples of 100 L were collected through an impaction method with a flow rate of 140 L/min (Millipore air Tester, Millipore, Billerica, MA, USA) onto each plate according to manufacturer’s instructions. In this case, air samples consisted mainly of 53 indoor samples and one outdoor sample collected in each bakery, which was to be used as a reference. All indoor samples were collected from workplaces occupied per one or two workers. Two different culture media were used in order to enhance the selectivity for fungal population growth: malt extract agar (MEA) supplemented with chloramphenicol (0.05%) and dichloran glycerol (DG18) agar-based media (Figure 1).

Air samples of 600 L were collected additionally using the impinger method. Samples were collected onto 10-mL sterile phosphate-buffered saline (PBS) with 0.05% Triton X-100, as reported previously [10]. 

For surface samples, the floors (considered as the most critical surface) of the same indoor locations (production, warehouse and store) were swabbed using a 10 by 10 cm square [10]. Settled dust and EDC sampling approaches were collected and extracted following the procedures previously described [23].

### 2.3. Fungal Contamination Characterization

All collected samples (air samples by impaction, surfaces samples, settled dust and EDC) were incubated at 25 ± 2 °C for 5–7 days (fungi). Fungal densities (colony forming units (CFU) per m^3^, m^2^ or g) were calculated on both MEA and DG18 media, followed by fungal identification achieved through macro and microscopic characteristics [25].

When colony overgrowth was observed due to fungi with fast growing rates (Mucorales group; *Chrysonilia sitophila* and *Trichoderma* spp.), making it impossible to count colonies, a quantitative cut off was applied of 500 isolates (CFU) as in previous published studies [10,26].

### 2.4. Molecular Detection of Specific Aspergillus Sections

Molecular identification of the different fungal species/strains was achieved by Real Time PCR (qPCR) using the Via 7 Real-time PCR System (Applied Biosystems) on air samples collected by the impinger method, surface swabs, settled dust and EDC (*n* = 27). Reactions and procedures were the same previously published [10] (Table 2).

### 2.5. Mycotoxins Analyses

Fifty-three air and 11 settled dust samples were screened for mycotoxins presence. Air samples (100 μL) were directly diluted 1:7 (*v*/*v*) with a 1:1 mixture of extraction solvent (acetonitrile (ACN): water (H_2_O): acetic acid (AcOH) (79:20:1)) and water. Settled dust samples (0.25 g) were extracted with 1 mL ACN: H_2_O: AcOH (79:20:1) for 60 min. Raw extracts were diluted with the same amount of water, mixed and filtered for mycotoxins detection. Detection of mycotoxins was carried out using high performance liquid chromatograph (HPLC) Nexera (Shimadzu, Tokyo, Japan) with a mass spectrometry detector API 4000 (Sciex, Foster City, CA, USA). Mycotoxins were separated on a chromatographic column Gemini NXC18 (150 × 4.6 mm, 3 μm) (Phenomenex, Torrance, CA, USA); mobile phase (A: water + 5 mM ammonium acetate + 1% acetic acid, B: methanol + 5 mM ammonium acetate + 1% acetic acid) mobile phase flow rate: 0.75 mL/min, injection volume: 7 μL.

Several mycotoxins were considered in the assessment performed, namely: patulin, nivalenol, deoxynivalenol-3-glucoside, deoxynivalenol, fusarenon-X, α-zearalanol, β-zearalanol, β-zearalenol, α-zearalenol, zearalanone, zearalenone, T2 tetraol, deepoxydeoxynivalenol, neosolaniol, 15-acetyldeoxynivalenol, 3-acetyldeoxynivalenol, monoacetoxyscirpenol, diacetoxyscirpenol, aflatoxin M1, aflatoxin B1, aflatoxin B2, aflatoxin G1, aflatoxin G2, fumonisin B1, fumonisin B2, fumonisin B3, T2 triol, roquefortine C, griseofulvin, T2 toxin, HT2 toxin, ochratoxin A, ochratoxin B, mycophenolic acid, mevinolin. Different Limits of Detection (LOD) and Quantification (LOQ) were obtained for each mycotoxin (Table 3).

### 2.6. Statistical Analysis

Data were analyzed using SPSS statistical software for Windows. The results were considered significant at the level of 5%. To test the normality of data the Shapiro-Wilk test was used. Regarding sample characterization frequency analysis (n; %) was performed for qualitative data and the calculation of the minimum and maximum for quantitative data was applied. The Kruskal-Wallis test was employed to compare the concentration of fungi (in air, surface and EDC) between bakeries (from 1 to 13), since the normality assumption was not verified. When statistically significant differences were detected, the Kruskal-Wallis multiple comparison test was used. The Spearman correlation coefficient was utilized to study the relationship between MEA and DG18 fungi concentration in different environmental matrices (air, surfaces and EDC), since the normality assumption was not verified.

## 3. Results

### 3.1. Fungal Characterization 

Fungal load in indoor air ranged from 0 to 2590 CFU·m^−3^ using MEA, with Bakery 4 presenting the highest median value (1053 CFU·m^−3^), followed by Bakeries 5, 1, 13 and 12 with mean values of 971, 690, 515 and 427 CFU·m^−3^, respectively. Noteworthy, 65.3% (32 out of 49) of the sampling sites showed higher fungal load than the limits imposed by the World Health Organization (WHO) (maximum value of 150 CFU·m^−3^) [30] in 10 bakeries the indoor air load mean was higher than the outdoor fungal load (Figure 1) and 30 out of the 49 (61.2%) air samples collected presented higher fungal load when compared to the outdoor sampling. 

Similar results were found obtained in DG18, with fungal load ranging from 0 to 2620 CFU·m^−3^ and with Bakery 4 presenting the highest median value (1108 CFU·m^−3^), followed by Bakeries 1, 11, 8 and 12, with mean values of 887, 550, 518 and 480 CFU·m^−3^, respectively. DG18 revealed that 62.3% of the sampling sites with fungal load exceeded the WHO limit; the indoor air load mean in eight bakeries was higher than the outdoor fungal load (Figure 1); and 47.2% of the air samples presented a higher indoor fungal load when compared to the outdoor sampling. 

Eighteen different fungal species/sections were found in air samples using MEA and 21 species/sections on DG18. *Cladosporium* spp. was the most prevalent in indoor air samples in both media (29.7% MEA; 48.7% DG18), followed by *Penicillium* spp. (22.3% MEA; 30.5% DG18) (Table 4). In addition to these species, *Alternaria* spp., *Aureobasidium* spp., *Aspergillus* sections (*Candidi*, *Circumdati* and *Nigri*), *Chrysonilia* sp., *Chrysosporium* spp., *Fusarium graminearum*, *Geotrichum* spp., Mucorales order (*Mucor* and *Rhizopus* genera), *Paecilomyces* spp., *Trichoderma* spp. *Ulocladium* spp. and *Verticillium* spp. were isolated as well utilizing MEA. Regarding DG18, in addition to the most prevalent species already mentioned, *Aspergillus* sections (*Aspergilli, Fumigati* and *Versicolores*), *Syncephalastrum racemosum* (Mucorales order) and *Trycothecium roseum* were identified.

Fungal load in surface swabs samples and EDC was distributed as follows: 0 to 51 × 10^5^ CFU·m^−2^ (MEA) and 0 to 101.3 × 10^5^ CFU·m^−2^ (DG18) and 0 to 3135 CFU·m^−2^ (MEA) and 0 to 4180 CFU·m^−2^ (DG18), respectively. Ten different fungal species/sections were found in surface swab samples on MEA and 13 on DG18 with *C sitophila* the most prevalent (75.8% MEA; 51.6% DG18). In addition *Aspergillus* sections (*Candidi* and *Versicolores*), *Chrysosporium* spp., *Phoma* spp., *Rhizopus* spp. (Mucorales order) and *Scopulariopsis brevicaulis* were identified with MEA, while *Aspergillus* sections (*Aspergilli*, *Candidi*, *Circumdati, Nigri* and *Versicolores*), *Chrysosporium* spp., *Geotrichum* spp., Mucorales order (*Mucor* spp. and *Rhizopus spp.*) and *Phoma* spp. were found with DG18.

With the use of EDC, it was possible to identify 11 different species/sections with MEA and 9 with on DG18. *C. sitophila* (91.9%) was the species most detected in EDC using MEA, whereas *Cladosporium* spp. (55.8%) and *Penicillium* spp. (38.3%) were the most noted with DG18 (Table 4). In addition to the most prevalent species, it was possible to isolate *Aspergillus* sections (*Aspergilli*, *Candidi*, *Circumdati*, *Flavi* and *Fumigati*,), *F. culmorum*, *Paecilomyces* spp. and *Syncephalastrum racemosum* (Mucorales order) on MEA plates and *Acremonium* spp. and *Paecilomyces* spp. on DG18 plates.

Settled dust did not show any fungal growth.

*Aspergillus* genera was detected in air samples using MEA and DG18 (0.3% and 1.3%, respectively). *Aspergillus* section *Candidi* was the most prevalent (62.5%) with MEA followed by *Nigri* (25%), whereas *Aspergillus* sections *Candidi* and *Circumdati* (35.5%) were more prevalent on DG18 followed by *Aspergilli* (22.6%). 

*Aspergillus* genera was found with MEA and DG18 (1.6% and 3%, respectively) on surface swabs samples. *Aspergillus* section *Candidi* and *Aspergillus* section *Versicolores* were the most prevalent species using MEA (50% each), whereas *Aspergillus* section *Versicolores* (88.5%) was more prevalent with DG18 followed by *Aspergillus* section *Candidi* (5.7%). 

Regarding EDC, *Aspergillus* genera was observed with MEA and DG18 (0.4% and 2.3%, respectively). *Aspergillus* section *Candidi* was the most prevalent using MEA (50%) followed by *Aspergillus* section *Circumdati* (25%), whereas *Aspergillus* sections *Fumigati* (50%) was more prevalent with DG18 followed by *Aspergillus* sections *Candidi* (33.3%) (Table 5).

Molecular detection—by real time PCR—of the target *Aspergillus* sections *Flavi* and *Circumdati* was negative for all environmental matrices analyzed. However, it was possible to detect *Aspergillus* section *Fumigati* in 22.4% on air samples by impinger samples, 27.8% on surface swabs and in 7.4% on EDC samples and *Aspergillus* section *Versicolores* on one air sample. In any of the samples detected, the target fungal sections/species had growth observed for the same sections/species.

### 3.2. Mycotoxins Contamination

None of the 36 mycotoxins were detected in air samples. Regarding settled dust, all samples showed contamination with 6 to 8 mycotoxins in each sample. The mycotoxins detected are presented in Table 6. DON was clearly the mycotoxin measured in higher amounts as all the samples identified quantifiable results (Table 6).

### 3.3. Comparison Analyses

**Figure 2:**
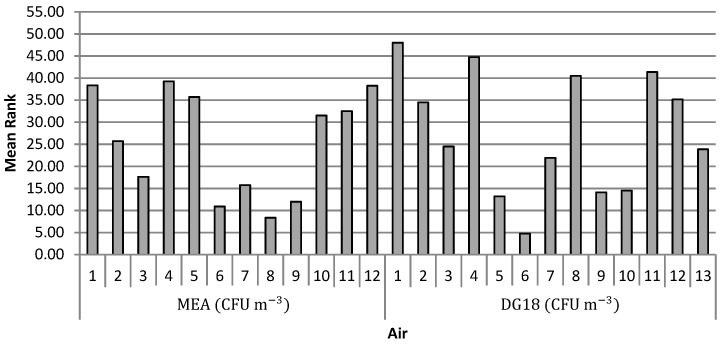
Mean ranks of fungal load (CFU m^−3^) on MEA and DG18 on the air in each bakery.

Significant differences of fungal load were detected in air samples and on surface swabs either with MEA or DG18. The multiple comparisons test Kruskal-Wallis which was applied to air samples, showed that the bakeries that differed significantly using MEA were Bakeries 7, 9 and 10 of Bakeries 1, 4, 5 and 13. It was found as well that Bakeries 7, 8 and 10 presented the lowest concentrations of fungal load (Figure 2). As for DG18, the bakeries that differed significantly were Bakeries 5, 6, 9 and 10 of 1, 4, 8 and 11. Bakeries 5, 6, 9 and 10 were the ones with lower concentrations (Figure 2). 

**Figure 3:**
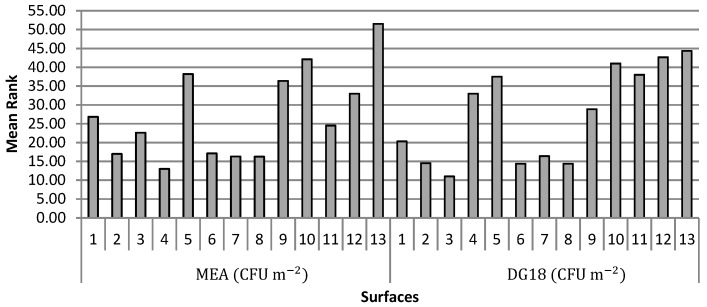
Mean ranks of fungal load (CFU m^−2^) on MEA and DG18 on the surface swabs in each bakery.

Regarding the surfaces, the load on MEA from Bakeries 4, 2, 6, 7 and 8 differed significantly from Bakeries 10 and 5 with the latter having the highest fungal load. As for the surfaces load using DG18, Bakeries 2, 3, 6, 7 and 8 differed from Bakeries 13, 12 and 10 indicating that Bakeries 2, 3, 6, 7 and 8 were the ones with the lowest fungal load (Figure 3). 

**Figure 4:**
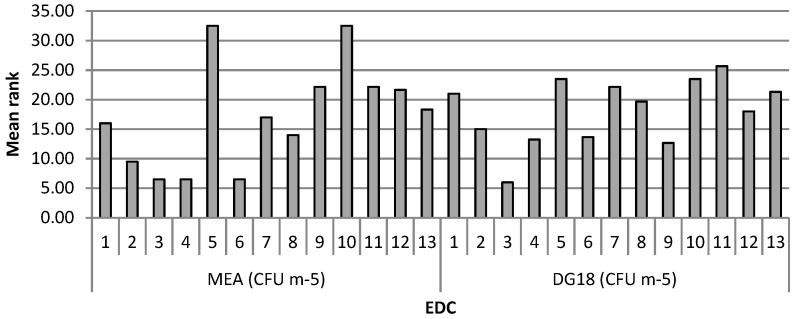
Mean ranks of fungal load (CFU m^−2^) on MEA and DG18 on EDC in each bakery.

The fungal load with MEA from EDC in Bakeries 2, 3, 4 and 6 differed significantly from Bakeries 5 and 10, which had a higher load (Figure 4). 

Significant positive and weak intensity correlations were detected between: (a) air fungal load with MEA and surface swab load with DG18; (b) the surface swab load with MEA with surface swab load on DG18 the EDC fungal load on MEA surface swab load on DG18 (r_S_ = 0.584, *p* = 0.000); (c) the surface swab load on DG18 with the EDC fungal load on MEA; (d) EDC fungal load on MEA with EDC fungal load on DG18. These results indicate that the higher load of some sampling methods in specific culture media is related to the higher load of others (sampling methods/specific culture media). 

A significant negative correlation with weak intensity, was detected between air fungal load on DG18 and surface fungal load on MEA. Data indicate that higher air fungal load on DG18 are related to lower surface load on MEA.

## 4. Discussion 

In this study, different sampling methods (active and passive), varying devices (impaction and impinger for active and surface swabs, settled dust and EDC for passive methods) and differing assays (culture-based with two different media and molecular tools) were applied. This multi-approach technique has advantages for the identification of *Aspergillus* sections *Flavi* and *Fumigati* on EDC by culture based-methods and detection of *Aspergillus* section *Fumigati* on air, surface swabs and EDC and *Aspergillus* section *Versicolores* on air by molecular tools that were not noted by culture-based methods. Indeed, complementarity of culture-based methods and molecular tools for occupational exposure assessments is well reported on literature overcoming the drawbacks of each other [22,23,26]. Further, different species were found in each media used (MEA and DG18), with an increased *Aspergillus* species identification on DG18, corroborating the findings already reported in different occupational environments [10]. These results reinforce the use of DG18 media, in addition to MEA as the default approach for microbiological analysis—even if the counts in both media correlated in some of the environmental matrices. Furthermore, DG18 was already reported as inhibitor of the spreading of Mucorales group, such as *Mucor* spp. and *Rhizopus* spp. and restrains the colony size of other fast-growing genera [31], allowing others, like *Aspergillus* genera, to grow. 

In addition, it was possible to detect mycotoxins on settled dust, whereas this was not the case on air sampling (active methods). In fact, assessing mycotoxins through passive methods (allowing the determination of the contamination levels of a greater period of time), such as settled dust or other environmental samples that collect contamination instead of load appears to be the trend for the mycotoxins contamination assessment in occupational environments [32,33,34].

Impinger air samples were exclusively used for fungal assessment by molecular tools as previous results [23] indicating that the impaction method was the best active sampling approach for the occupational exposure assessment of the viable bioburden in this specific occupational environment [23]. Of relevance, is the fact that 76.9% (10 out of 13) of the assessed bakeries exceeded the WHO guidelines in at least one of the applied media and 30 air samples on MEA, and presented a higher fungal load than outdoors, potentially indicating indoor sources of fungal contamination, such as workers and their activities which contribute to indoor airborne microbial load [35] as well as raw materials entering the facilities [6,8]. Among the 13 bakeries assessed, fungal species with toxigenic potential [36] were identified in all environmental matrices where fungal growth occur, including *Penicillium* spp., *Fusarium* spp. and *Aspergillus* sections. 

However, although it was not possible to observe or detect fungal growth in settled dust samples, several mycotoxins were present. Mycotoxins persist in an occupational environment even in fungi absence as they resist to adverse abiotic factors such as temperature [37,38] and this is the reason why lack of fungal growth cannot be used as a surrogate for non-detectable mycotoxins’ contamination [39]. Indeed, mycotoxins are commonly present in airborne dust [40,41] and in fungal spores or fragments of microbial growth, and both can act as mycotoxin carriers to the workers respiratory system, since mycotoxins are not volatile [42]. This is an important characteristic because—as already mentioned in this occupational setting—exposure to organic dust is commonly observed [43,44] and can enhance exposure to mycotoxins by inhalation. Thus, the most common scenario, found in other occupational settings as well, is the co-exposure to more than one risk factor—fungi and metabolites (mycotoxins) [10,26]. Overall, this multi-approach on sampling methods (active and passive) and lab assays (culture based-methods and molecular tools) enriched data findings, enabling a more detailed risk [10,22], and the identification of potential fungal and mycotoxin contamination sources (flour and other raw materials on settled dust composition). 

Limitations in this study was the potential influence of climacteric conditions on the seasonal distribution of fungal species, such as *Alternaria*, *Aspergillus*, *Cladosporium* and *Penicillium*, as some bakeries were sampled in the winter season and others during spring season [45]. Whereas the sample is constituted by only two bakery companies, the total comprised 13 bakeries exemplify a reliable picture of bakery industry in Portugal concerning workload, workplace layout, environmental parameters, production processes, and final food commodities produced. 

Considering the obtained results, further studies are needed to improve our understanding of the occupational environment of the bakeries. A previous study developed in a similar occupational setting—the milling industry [46]—using biomonitoring tools, did not demonstrate additional risk for workers related to occupational exposure to mycotoxins. However, different tasks, workplace conditions, production processes and even different outdoor climatic conditions might affect individuals regarding mycotoxins exposure. Indeed, a study developed in a fresh bread dough company located in Portugal showed that workplace exposure to specific mycotoxins added significantly to the exposure of mycotoxins occurring by ingestion [17], particularly in the case of deoxynivalenol. Moreover, the study demonstrated that both workers and controls are exposed to several mycotoxins simultaneously. Such exposure constitutes a large potential of associated health effects due to the synergistic and/or additive toxicities, which are not still completely understood scientifically [47]. Therefore, we should consider that the risk of a mixture typically exceeds the risk of each individual mixture component and second, even when it is guaranteed that all compounds of a mixture are present at concentrations that are considered safe from a health or regulatory perspective, the resulting mixture can still cause health effects [48]. More research work should be developed to elucidate the possible interactions among mycotoxins and to support the definition of new health-based guidance values for grouped mycotoxins and/or co-occurring mycotoxins in order to prevent human disease [47].

Considering the results obtained in this study, it is possible to understand that the fungal burden is an important component of the bakeries environment and, as mentioned before, can have a role in “baker’s asthma” as well. Future work should be developed to assess the impact on workers’ health of the simultaneous exposure to all the risk factors in presence (e.g., cereals’ flours, fungi and their metabolites) and to try to understand whether the mixture can play a role in “baker’s asthma” disease.

## 5. Conclusions

The multi-approach analysis based on the use of different sampling methods (active and passive), different sampling devices (impaction and impinger for active and surface swabs, settled dust and EDC for passive methods), and different assays allowed for a wider spectrum of the fungal burden in the assessed bakeries to be obtained. Thus, industrial hygienists and exposure assessors should rely on different sampling methods (active and passive) and different assays (culture based and molecular methods) to obtain an accurate risk characterization regarding fungal burden. The results obtained regarding fungal load and contamination emphasizes the increased exposure to this occupational risk, besides other relevant allergens commonly present in the environment, and the need for further studies to improve understanding of this setting and develop surveillance and intervention programs aimed at the improvement and protection of the respiratory health of bakery workers. The information regarding settled dust contamination by several mycotoxins was useful for the awareness for the presence of this occupational risk and to ponder the raw material (e.g., flour) as an indoor contamination source.

## Figures and Tables

**Table 1:** Environmental and raw material samples distributed for each bakery assessed for mycobiota analyses.

Bakery	Facilities	Sampling Approaches (Samples Number)				
		Indoor Air Sampling Impaction MEA and DG18	Indoors Air Sampling Impinger	Surface Swabs	Settled Dust #	EDC *
1	Enlarged company	3	3	3	-	2
2	Enlarged company	5	5	5	-	3
3	Enlarged company	4	4	4	1	2
4	Enlarged company	4	4	4	1	2
5	Enlarged company	5	5	5	1	3
6	Enlarged company	4^+^	4	4	1	3
7	Enlarged company	5	5	5	1	3
8	Enlarged company	4	4	4	1	3
9	Supermarket	4	4	4	1	3
10	Supermarket	4	4	5	1	3
11	Supermarket	4	4	4	1	3
12	Supermarket	3	3	3	1	3
13	Supermarket	4	4	4	1	3
Total		53	53	58	11	36

* Adopted from Viegas et al. [23]; # Adopted [24] + Samples impacted on MEA were not possible to perform.

**Table 2:** Sequence of primers and TaqMan probes used for Real Time PCR.

*Aspergillus* Sections Targeted		Sequences	Reference
***Flavi* (Toxigenic Strains)**	Forward Primer	5′-GTCCAAGCAACAGGCCAAGT-3′	[27]
	Reverse Primer	5′-TCGTGCATGTTGGTGATGGT-3′	
	Probe	5′-TGTCTTGATCGGCGCCCG-3′	
***Fumigati***	Forward Primer	5′-CGCGTCCGGTCCTCG-3′	
	Reverse Primer	5′-TTAGAAAAATAAAGTTGGGTGTCGG-3′	[28]
	Probe	5′-TGTCACCTGCTCTGTAGGCCCG-3′	
***Circumdati***	Forward Primer	5′-CGGGTCTAATGCAGCTCCAA-3′	
	Reverse Primer	5′-CGGGCACCAATCCTTTCA-3′	[26]
	Probe	5′-CGTCAATAAGCGCTTTT-3′	
***Versicolores***	Forward Primer Reverse Primer Probe	5′–CGGCGGGGAGCCCT-3′ 5′–CCATTGTTGAAAGTTTTGACTGATcTTA-3′ 5′–AGACTGCATCACTCTCAGGCATGAAGTTCAG-3′	[29]

**Table 3:** Limits of detection (LOD) and Quantification (LOQ) for mycotoxins analysed by LC-MS/MS.

Mycotoxins	Limit of Detection LOD (ng/g)	Limit of Quantification (LOQ) (ng/g)
Patulin	1.1	3.6
Nivalenol	4.5	14.9
Deoxynivalenol-3-glucoside	5.4	17.8
Deoxynivalenol	2.7	8.9
Fusarenon-X	4.8	15.8
Deepoxy-deoxynivalenol	4.2	13.9
α-Zearalanol	2.0	6.6
β-Zearalanol	0.9	3.0
β-Zearalenol	1.4	4.6
α-Zearalenol	1.0	3.3
Zearalanone	0.5	1.7
Zearalenone	0.2	0.7
T2 Tetraol	5.4	17.8
Deepoxydeoxynivalenol	0.4	1.3
Neosolaniol	0.1	0.3
15-Acetyldeoxynivalenol	0.8	2.6
3-Acetyldeoxynivalenol	0.8	2.6
Monoacetoxyscirpenol	0.1	0.3
Diacetoxyscirpenol	0.3	1.0
Aflatoxin M1	0.1	0.3
Aflatoxin B1	0.1	0.3
Aflatoxin B2	0.1	0.3
Aflatoxin G1	0.1	0.3
Aflatoxin G2	0.1	0.3
Fumonisin B1	0.5	1.7
Fumonisin B2	0.4	1.3
Fumonisin B3	0.5	1.7
T2 Triol	0.3	1.0
Roquefortine C	0.2	0.7
Griseofulvin	0.1	0.3
T2	0.1	0.3
HT2	0.3	1.0
Ochratoxin A	0.1	0.3
Ochratoxin B	0.1	0.3
Mycophenolic acid	0.2	0.7
Mevinolin	0.1	0.3

**Table 4:** Fungal distribution on the collected environmental samples after inoculation onto MEA and DG18 media.

MEA		DG18	
**Air**	**(CFU·m^−3^) (%; n)**	**Air**	**(CFU·m^−3^) (%; n)**
*Acremonium* sp. *Chrysonilia sitophila* *Cladosporium* sp. Mucorales order *Penicillium* sp. Others	(17.0; 3960) (17.2; 4000) (29.7; 6920) (2.2; 520) (22.3; 5190) (11.7; 2730)	*Acremonium* sp. *Chrysonilia sitophila* *Cladosporium* sp. *Geotrichum* sp. *Penicillium* sp. Others	(2.8; 670) (10.3; 2500) (48.7; 11860) (2.5; 620) (30.5; 7420) (5.2; 1270)
**Surfaces**	**(CFU·m^−2^) (%; n)**	**Surfaces**	**(CFU·m^−2^) (%; n)**
*Acremonium* sp. *Chrysonilia sitophila* *Cladosporium* sp. *Penicillium* sp. Others	(7.7; 2040000) (75.8;20000000) (10.7; 2820000) (4.0; 1050000) (1.8; 480500)	*Acremonium* sp. *Chrysonilia sitophila* *Cladosporium* sp. Mucorales order *Penicillium* sp. Others	(4.7; 1370000) (51.6;15020000) (10.5; 3070000) (17.5; 5110000) (12; 3500000) (3.6; 1060000)
**EDC**	**(CFU·m^−2^) (%; n)**	**EDC**	**(CFU·m^−2^) (%; n)**
*Chrysonilia sitophila* *Cladosporium* sp. *Penicillium* sp. Others	(91.9; 124403) (2.5; 3434) (5.0; 6718) (0.6; 746)	*Aspergillus* sp. *Chrysosporium* sp. *Cladosporium* sp. *Penicillium* sp. Others	(2.3; 597) (3.1; 796) (55.8; 14480) (38.3; 9952) (0.5; 149)

**Table 5:** *Aspergillus* sections’ distribution in the collected samples.

MEA		DG18	
**Air**	**(CFU·m^−3^) (%; n)**	**Air**	**(CFU·m^−3^) (%; n)**
*Candidi* *Circumdati* *Nigri*	(62.5; 50) (12.5; 10) (25; 20)	***Aspergilli*** ***Candidi*** ***Circumdati*** ***Fumigati*** ***Nigri*** ***Versicolores***	(22.6; 50) (35.5; 110) (35.5; 110) (3.2; 10) (3.2; 10) (3.2; 10)
**Surfaces swabs**	**(CFU·m^−2^) (%; n)**	**Surfaces swabs**	**(CFU·m^−2^) (%; n)**
*Candidi* *Versicolores*	(50; 210000) (50; 210000)	***Aspergilli*** ***Candidi*** ***Circumdati*** ***Nigri*** ***Versicolores***	(1.1; 10000) (5.7; 50000) (2.3; 20000) (2.3; 20000) (88.5; 870000)
**EDC**	**(CFU·m^−2^) (%; n)**	**EDC**	**(CFU·m^−2^) (%; n)**
***Aspergilli*** ***Candidi*** ***Circumdati*** ***Flavi*** ***Fumigati***	(8.3; 50) (50; 299) (25; 149) (8.3; 50) (8.3; 50)	*Aspergilli* *Candidi* *Fumigati* *Versicolores*	(8.3; 50) (33.3; 199) (50; 299) (8.3; 50)

**Table 6:** Mycotoxins contents (>LOD) in settled dust (ng/g).

Mycotoxins	Number of Samples with Detectable Values	Concentration Range
**DON**	12 (100%)	15.95–211
**D3G**	9 (75%)	<LOQ–25.77
**ZEA**	12 (100%)	<LOQ–1.60
**15-ADON**	3 (25%)	5.01–5.22
**MAS**	6 (50%)	0.40–1.6
**DAS**	1 (8.3%)	<LOQ
**FB1**	3 (25%)	5.23–7.36
**FB2**	4 (33.3%)	3.73–5.49
**GRIS**	1 (8.3%)	5.90
**HT2**	2 (16.6%)	0.99–1.93
**OTA**	10 (83.3%)	<LOQ–0.64
**OTB**	1 (8.3%)	0.54
**MPA**	7 (58.3%)	<LOQ–3.04
